# MELK aggravates lung adenocarcinoma by regulating EZH2 ubiquitination and H3K27me3 histone methylation of LATS2

**DOI:** 10.1111/jcmm.18216

**Published:** 2024-04-23

**Authors:** Hui Yu, Xianrong Xu, Lirong Zhu, Shengjie Chen, Jincheng He

**Affiliations:** ^1^ Department of Thoracic Surgery Affiliated Hospital of Jiangsu University Zhenjiang China; ^2^ Department of Respiratory and Critical Care Medicine Affiliated Hospital of Jiangsu University Zhenjiang China; ^3^ Surgical Department Danyang Maternal and Child Health Hospital Danyang China

**Keywords:** degradation, EZH2, H3K27me3, LATS2, lung adenocarcinoma, MELK, methylation modification, proliferation invasion, ubiquitination

## Abstract

We tried to elucidate the possible roles of maternal embryonic leucine pull chain kinase (MELK) in lung adenocarcinoma (LUAD) growth and metastasis. Differentially expressed genes in LUAD samples were analysed by the GEPIA database. Clinical tissue samples and cells were collected for MELK, EZH2 and LATS2 expression determination. Co‐IP assay was used to verify the interaction between EZH2 and MELK; CHX tracking assay and ubiquitination assay detected the degradation of MELK on EZH2 ubiquitination. ChIP assay detected the enrichment of EZH2 and H3K27me3 on the LATS2 promoter region. LUAD cells were selected for in vitro validation, and the tumorigenic ability of LUAD cells was also observed in a transplantation tumour model of LUAD nude mice. MELK and EZH2 were highly expressed in LUAD samples, while LATS2 was lowly expressed. MELK interacted with EZH2 to inhibit its ubiquitination degradation; EZH2 elevated H3K27me3 modification in the LATS2 promoter to lower LATS2 expression. Silencing MELK or EZH2 or overexpressing LATS2 restrained LUAD cell proliferation and invasion, and facilitated their apoptosis. Silencing MELK or EZH2 or overexpressing LATS2 suppressed tumour formation in nude mice. This study demonstrated that MELK aggravated LUAD by upregulating EZH2 and downregulating LATS2.

## 1 INTRODUCTION

Lung adenocarcinoma (LUAD), one of the main subtypes of non‐small‐cell lung carcinoma (NSCLC), has a shorter survival time than patients with other subtypes of NSCLC.^1^, ^2^ LUAD develops from small airway epithelial, type II alveolar cells, which release mucus and other substances.^3^ The well‐established high risk of metastasis and invasiveness associated with LUAD leads to a dismal 5‐year survival rate of only 19%.^4^ Despite significant advancements in recent years in the primary therapeutic approaches, such as molecular targeted therapy, radiation, chemotherapy and surgical resection, the prognosis of LUAD patients remains dismal.^5^, ^6^ In order to identify and validate particular biomarkers and treatment targets for LUAD, special investigations are needed.

Maternal embryonic leucine zipper kinase (MELK), also named MPK38, belongs to the AMP‐activated protein kinase family of serine–threonine kinases and was first detected in Xenopus oocytes and embryos.^7^ Intriguingly, MELK is an attractive therapeutic target for the modulation of cancers.^8^ Moreover, the tumour‐promoting potential of MELK has been reported in LUAD.^9^ Notably, MELK can bind to and phosphorylate EZH2 to elevate EZH2 expression, thus affecting medulloblastoma cancer stem‐like cell proliferation.^10^ As the enzymatic catalytic subunit of PRC2, EZH2 can coordinate the expression of downstream target genes through trimethylation of H3K27me3.^11^ Importantly, evidence supports that EZH2 can be recruited into the promoter of LATS2 to augment H3K27me3 methylation, thus restraining LATS2 expression in fulminant hepatic failure.^12^ Interestingly, EZH2 is demonstrated to promote LUAD development.^13^ Besides, LATS2 is able to inhibit the progression of LUAD.^14^ Herein, from the aforementioned findings, this research was expected to contribute a novel target for LUAD treatment based on the effects of MELK with the involvement of the EZH2/LATS2 axis.

## 2 MATERIALS AND METHODS

### 2.1 Ethics statement

The Ethics Committee of the Affiliated Hospital of Jiangsu University provided Ethical Approval for the experiments involving human beings in this study. This study was supported and agreed by the patients via signed informed consent. Animal experiments were implemented under the ratification of the Animal Ethics Committee of the Affiliated Hospital of Jiangsu University.

### 2.2 Bioinformatics analysis

The gene expression in normal tissues and LUAD tissues from TCGA and GTEx was analysed by the GEPIA database.

The ChIP‐seq microarray data GSE76626 of EZH2‐deficient human embryonic stem cells were obtained from the Gene Expression Omnibus (GEO) database. The expression of H3K27me3, H3K27ac, H3K4me1 and H3K4me3 in the gene's promoter region was analysed.

### 2.3 Clinical samples

A total of 31 patients with LUAD (18 males and 13 females, age: 41 ~ 73 years, mean age: 56.84 ± 8.42 years) who underwent surgery from June 2016 to June 2017 were selected. Fresh LUAD tissues and adjacent tissues (at least 5 cm from cancer lesions) were obtained by surgery. The obtained tissues were immediately frozen in liquid nitrogen and stored in a refrigerator at −80°C until the beginning of the experiment. The clinicopathological features of all patients, such as age, sex, tumour size, in situ infiltration, distant metastasis and tumour‐node‐metastasis stage, were obtained. The patients were followed up from the end of the operation to June 2020 (lasting 4–36 months) to complete the clinical data. MELK expression was quantified utilizing RT‐qPCR. The Kaplan–Meier method was adopted to assess the correlation between MELK expression and overall survival (OS) of LUAD patients.

### 2.4 Cell culture and transduction

Three kinds of LUAD cell lines A549 (CRM‐CCL‐185, ATCC, VA), H1975 (CRL‐5908, ATCC) and PC‐9 (CL‐0668, Procell Life Science & Technology Co., Ltd., Wuhan, China) and human bronchial epithelial cells 16HBE (CL‐0249, Procell Life Science & Technology) were cultured in Dulbecco's Modified Eagle Medium (11,965,092, Gibco, Carlsbad, California) appended to 10% fetal bovine serum (FBS, 16140071, Gibco), 10 μg/mL streptomycin and 100 U/mL penicillin (15,140,163, Gibco) and placed in an incubator with 5% CO_2_ at 37°C.

Cells were seeded into the 6‐well plate with 1 × 10^5^ cells/well. After incubation for 24 h, upon cell confluence reached about 75%, cells were transduced with sh‐NC, sh‐MELK, sh‐EZH2, sh‐LATS2, sh‐MELK + sh‐NC, sh‐EZH2 + sh‐NC or sh‐MELK + sh‐LATS2 utilizing Lipofectamine 2000 (11,668,019, Invitrogen Inc., Carlsbad, CA). The follow‐up experiment was carried out after transduction for 48 h.

The silencing sequences are described in Table S1. The silencing plasmids (concentration of 50 ng/mL) were procured from GenePharma (Shanghai, China). The EPZ‐6438 (EZH2 inhibitor, SC0056, Beyotime Institute of Biotechnology, Shanghai, China, 0.02 μM), MG132 (M7449, Sigma‐Aldrich, St. Louis, MO, 10 μM) and cycloheximide (CHX, ab120093, Abcam Inc., Cambridge, United Kingdom, 10 μg/mL) were applied.

### 2.5 RT‐qPCR

The total RNA was extracted by Trizol (15,596,026, Invitrogen). The RNA was reversely transcribed into cDNA with the help of a PrimeScript RT reagent Kit (RR047A, Takara Holdings Inc., Kyoto, Japan). The synthesized cDNA was checked through RT‐qPCR with the help of Fast SYBR Green PCR kit (4,364,344, Applied Biosystems, Carlsbad, CA) and ABI PRISM 7500 qPCR system (Applied Biosystems). The $2^{-\Delta \Delta C_{t}}$ method was utilized to analyse the relative expression of MELK, EZH2 and LATS2 genes with β‐actin as an internal reference. The primer design is described in Table S2.

### 2.6 Immunoblotting

Immunoblotting was completed with diluted primary antibodies purchased from Abcam to rabbit anti‐MELK (ab273015, 1:1000), rabbit anti‐EZH2 (ab186006, 1:1000), rabbit anti‐LATS2 (ab110780, 1:1000), mouse anti‐β‐actin (ab8226, 1:1500), rabbit anti‐Fas (ab133619, 1:1000), mouse anti‐proliferating cell nuclear antigen (PCNA; ab29, 1:1500) and rabbit anti‐matrix metalloproteinase (MMP, ab51075, 1:1000) and antibody purchased from Cell Signalling Technologies (Beverly, MA) to rabbit anti‐hemagglutinin (HA, 3724, 1:1000) as well as with horseradish peroxidase‐tagged goat anti‐rabbit (ab205718, 1:2000, Abcam) and goat anti‐mouse (ab6789, 1:2000, Abcam) secondary antibodies. The membrane was developed with an enhanced chemiluminescence working solution (WBKLS0050, EMD Millipore, Billerica, MA). ImageJ analysis software was run to quantify the grey value of each band with β‐actin as the internal reference.

### 2.7 CCK‐8 assay

The cells were seeded in a 96‐well plate (1 × 10^3^ cells/well) for incubation in the incubator overnight to make the cells adhere to the wall. Then, 10 μL CCK‐8 reagent (C0038, Beyotime) was added to the culture medium. After about 4 h, the absorbance at the wavelength of 450 nm was measured by microplate. A line chart was drawn according to the absorbance of different periods.

### 2.8 Transwell experiment

The upper surface of the bottom membrane of the Transwell chamber was coated with Matrigel (E1270, Sigma‐Aldrich) to assess cell invasion capability. The stained invasive cells were counted under the orthostatic optical microscope (Primotech, CarlZeiss, Oberkochen, Germany) with five visual fields selected randomly.

### 2.9 Flow cytometry

The Annexin V‐fluorescein isothiocyanate (FITC) apoptosis kit (556,419, Becton, Dickinson and Company, NJ) was used for the experiment. The cells were collected, then gently suspended with PBS and counted. The 5–10 × 10^4^ resuspended cells were centrifuged at 200*g* for 5 min. After removal of the supernatant, 195 μL Annexin V‐FITC was added to resuspend the cells, which were then incubated with 5 μL Annexin V‐FITC at 20°C–25°C without light for 10 min and with 10 μL propidium iodide staining solution avoiding light exposure. The cells were measured by flow cytometry (651,154, Becton, Dickinson and Company).

### 2.10 Co‐IP

Cells were washed twice with pre‐cooled PBS and centrifuged at 4°C and 14,000 g for 15 min. Bradford method was used to make a protein standard curve and determine protein concentration. The total protein was diluted to about 1 μg/μL with PBS. Then, the total protein (500 μL) was added with 1 μL rabbit anti‐MELK (ab273015, Abcam). The NC group added 1 μL rabbit anti‐IgG (ab172730, Abcam). After that, 100 μL protein A agarose beads were added to capture antigen–antibody complexes. After centrifugation at 5000 rpm for 2 min, agarose beads‐antigen antibody complexes were collected. Then, the complex was washed thrice with pre‐cooled RIPA buffer, suspended with 60 μL 2 × loading buffer and mixed gently. The samples were boiled for 5 min, and the following steps were the same as immunoblotting.

### 2.11 CHX tracking experiment

Cells transduced with PCDH, PCDH‐MELK, sh‐NC and sh‐MELK were inoculated into a 6‐well plate separately. These cells were cultured with 10 μg/mL CHX and harvested at 0, 60 and 120 min respectively. The cells were washed twice with pre‐cooled PBS, added with pre‐cooled RIPA buffer and centrifuged at 4°C and 14,000 g for 15 min with the supernatant obtained. The protein was diluted to 1 μg/μL with PBS and boiled in a metal bath at 100°C for 10 min. The protein concentration was determined by immunoblotting.

### 2.12 Ubiquitination experiment

EZH2 was transfected into cells by a protein ubiquitination kit (#BK161, the microtubule cytoskeleton. Proc. Natl. Acad. Sci). After 24 h, the target protein was extracted and separated. EZH2 was incubated with HA‐ubiquitin and MELK in a buffer in vitro. Then, the products were analysed by Co‐IP and immunoblotting.

### 2.13 ChIP

ChIP was completed employing the EZ‐Magna ChIP TMA kit (17–408, Sigma‐Aldrich). In the experimental group, the supernatant was mixed with 1 μL rabbit anti‐EZH2 (ab186006, Abcam) and H3K27me3 (ab6002, Abcam) respectively. In the NC group, the supernatant was added with 1 μL rabbit anti‐IgG (ab730, Abcam). The DNA was recovered by decross‐linking with 20 μL 5 M NaCl. The enriched chromatin fragments were evaluated by PCR. RT‐qPCR was adopted to assess the enrichment of EZH2 and H3K27me3 in the LATS2 promoter region. LATS2 promoter primer (F: 5'‐ACATCTTTGAAGGCAGAGCAGGA‐3′, R: 5'‐TAAGCTGACTATACTTTCTCCACCC‐3′).

### 2.14 Establishment of a xenograft tumour model in nude mice

A total of 36 healthy female nude mice aged 4 weeks old (weighing 23 ± 2 g) were individually housed in a specific pathogen‐free animal laboratory (22°C ~ 25°C temperature, 60% ~ 65% humidity and a 12‐h light/dark cycle). The mice were given ad libitum access to food and water. The experiment was conducted after acclimation for 1 week.

During the feeding process, nude mice were injected with cells stably transduced with sh‐NC, sh‐MELK + sh‐NC, sh‐EZH2 + sh‐NC or sh‐MELK + sh‐LATS2 plasmids through the medial side of the right forelimb with 2 × 10^7^ cells/mice (*n* = 9). The tumorigenesis of nude mice was observed on the 7th, 14th, 21st and 28th day after subcutaneous injection. A vernier calliper was used to measure and record the long diameter (a) and short diameter (b) of the tumour. The tumour volume was calculated as = (a × b^2^)/2 and the tumour growth curve was drawn. After further feeding for 4 weeks, nude mice were weighed every day. After 4 weeks, the mice were killed for detection of the tumour size and weight.

### 2.15 Statistical analysis

SPSS 21.0 (IBM Corp. Armonk, NY) was adopted for statistical analysis. The measurement data were summarized as mean ± standard deviation. Unpaired *t*‐test, one‐way analysis of variance (ANOVA) with Tukey's post hoc test, repeated measures ANOVA with Bonferroni post hoc test and two‐way ANOVA were applied for data comparison. Kaplan–Meier was employed to calculate the survival rate of patients. A log‐rank test was implemented for univariate analysis. *p* < 0.05 was deemed as a significant difference.

## 3 RESULTS

### 3.1 MELK is highly expressed in LUAD tissues and cells, which is associated with poor prognosis

**FIGURE 1:**
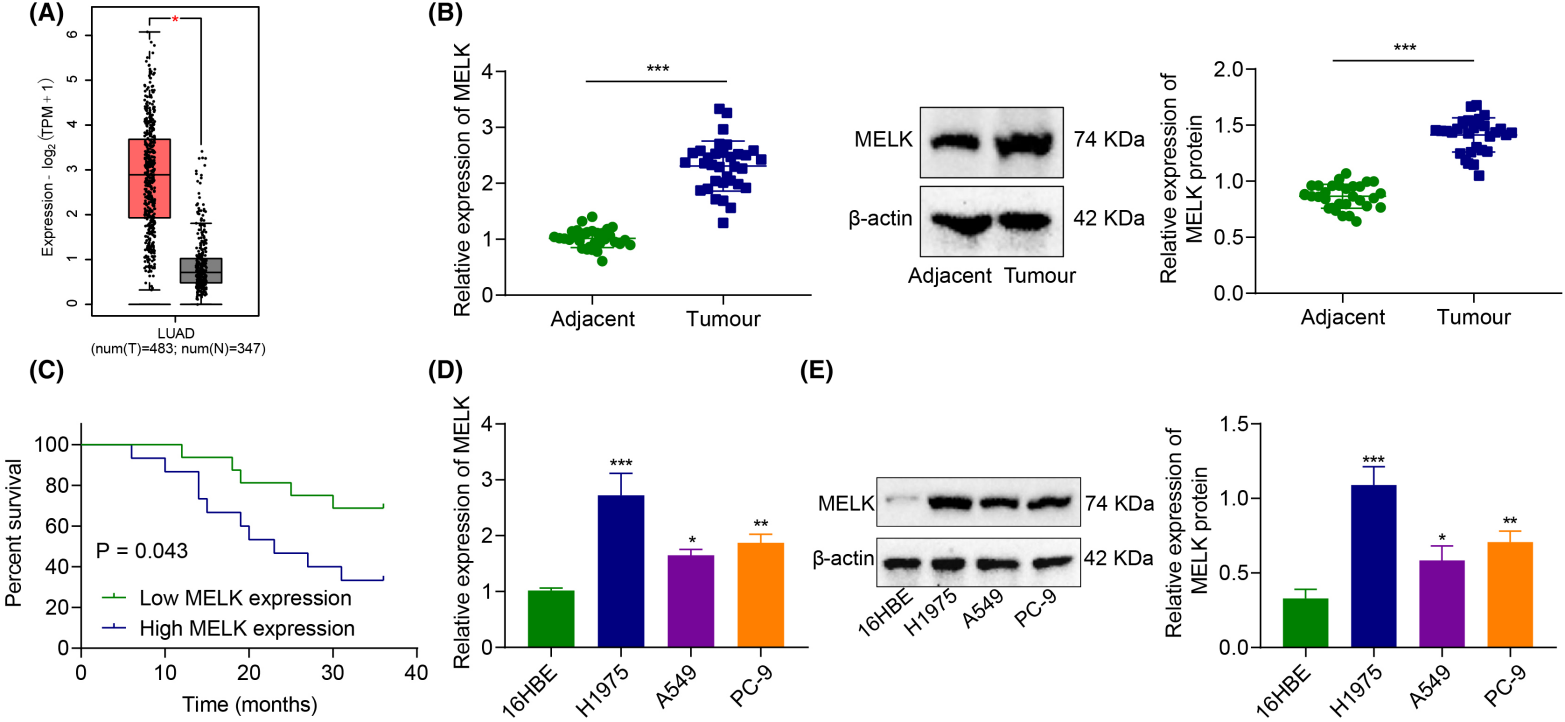
MELK is upregulated in LUAD tissue and cells and is correlated with poor prognosis. (A) mRNA level of MELK gene in GEPIA database. Red indicates tumour tissue, and grey indicates normal tissues (*p < 0.05 vs. normal tissues). (B) The expression of MELK in LUAD tissues and adjacent tissues from 31 patients with LUAD detected by RT‐qPCR and western blot analysis (***p < 0.001 vs. adjacent tissues). (C) Kaplan–Meier method to analyse the correlation of MELK expression with OS of LUAD patients. (D) The mRNA level of MELK detected by RT‐qPCR. (E) The protein level of MELK determined by western blot analysis. *p < 0.05, **p < 0.01 and ***p < 0.001 compared with 16HBE cells. The cell experiment was repeated three times.

A prior study has revealed that MELK acts as an oncogenic gene,^15^ but its downstream regulatory mechanism remains unclear. GEPIA analysis depicted that MELK was upregulated in LUAD tissues (Figure 1A).

RT‐qPCR and immunoblotting exhibited that MELK expression was higher in the LUAD tissues (vs adjacent tissues) (Figure 1B). The patients were grouped into patients with high MELK expression and patients with low MELK expression, with the median MELK expression in LUAD tissues as the boundary. The Kaplan–Meier method (Figure 1C) showed that the OS of patients with high MELK expression was lower than those with low MELK expression, highlighting that high MELK expression was associated with poor prognosis.

Meanwhile, compared with human bronchial epithelial cell 16HBE, MELK expression in LUAD cells (A549, H1975 and PC‐9) was markedly increased, with the highest expression in H1975 cells. Therefore, LUAD cell line H1975 was selected for subsequent experimentations (Figure 1D,E).

### 3.2 Silencing MELK inhibits LUAD cell malignant properties

**FIGURE 2:**
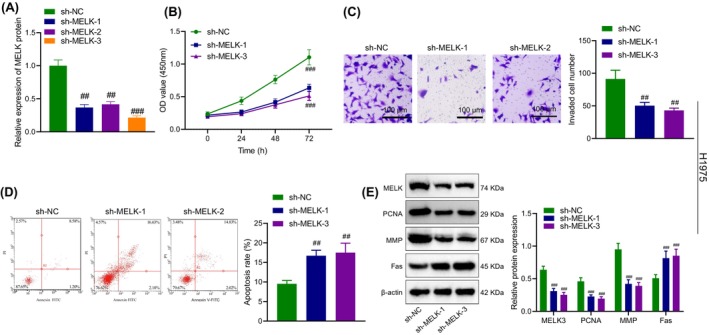
The effect of MELK silencing on proliferation, invasion and apoptosis of LUAD cell line H1975. (A) RT‐qPCR to detect MELK expression in H1975 cells; (B) CCK8 assay to detect cell proliferation ability of H1975 cells; (C) Transwell assay to detect cell invasion ability of H1975 cells; (D) Flow cytometry to detect cell apoptosis of H1975 cells; (E) Western blot to detect expression levels of related proteins in H1975 cells. ##*p* < 0.01 and ###*p* < 0.001 versus H1975 cells transduced with sh‐NC. The cell experiment was repeated three times.

To explore the effect of MELK on LUAD cells, MELK was silenced in H1975 and PC‐9 cells. Immunoblotting corroborated that MELK expression in H1975 and PC‐9 cells was reduced by treatment with sh‐MELK‐1, sh‐MELK‐2 or sh‐MELK‐3, especially sh‐MELK‐3. Thus, sh‐MELK‐3 was selected for the subsequent experimentations (Figure 2A).

As detected by CCK‐8, Transwell assay and flow cytometry (Figure 2B–D, Figure S1A–C), MELK knockdown restrained the proliferative and invasive abilities of H1975 and PC‐9 cells and enhanced apoptosis.

As reflected by immunoblotting, silencing MELK contributed to a decline in PCNA and MMP expression and an increase in Fas expression in H1975 and PC‐9 cells (Figure 2E, Figure S1D).

### 3.3 MELK promotes EZH2 stability by inhibiting the ubiquitination and degradation of EZH2 in LUAD cells

**FIGURE 3:**
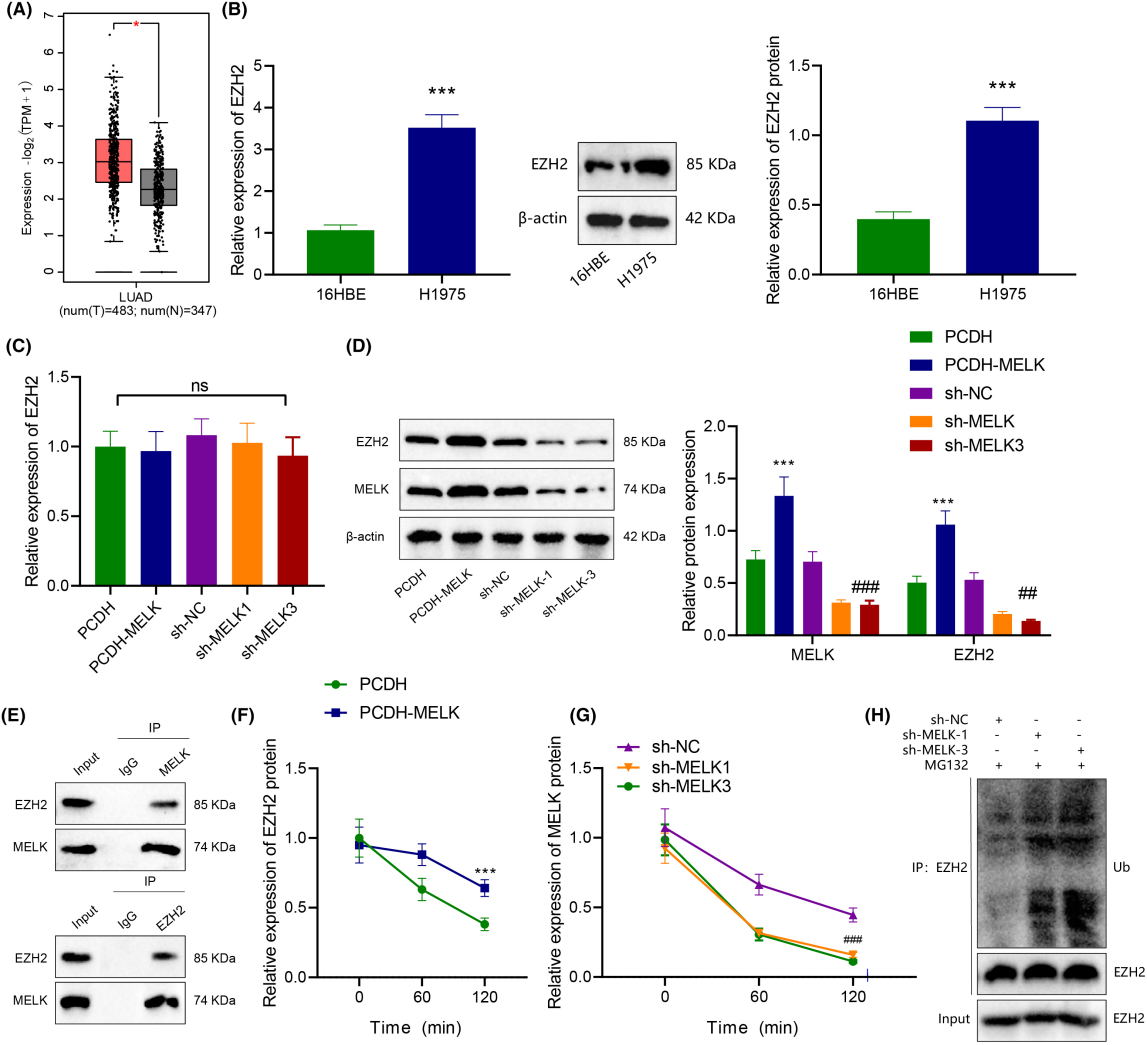
MELK increases EZH2 stability by curtailing EZH2 ubiquitination and degradation in LUAD cells. (A) EZH2 mRNA level in GEPIA database. Red indicates tumour tissue and grey indicates normal tissues (*p < 0.05 vs. normal tissues). (B) mRNA and protein levels of EZH2 in H1975 LUAD cells and human bronchial epithelial cells 16HBE measured by RT‐qPCR and western blot analysis. (C) The mRNA level of EZH2 in H1975 cells after overexpression and silencing of MELK determined by RT‐qPCR. (D) The expression of MELK and EZH2 in H1975 cells after overexpression and silencing of MELK measured by western blot analysis. (E) The interaction between MELK and EZH2 tested by Co‐IP. (F) The effect of overexpression of MELK on the stability of EZH2 in H1975 cells evaluated by CHX tracing assay. (G) The effect of silencing MELK on EZH2 stability in LUAD cells assessed by CHX tracing test. (H) The effect of silencing MELK on EZH2 ubiquitination modification evaluated by protein ubiquitination experiment. ***p < 0.001 versus 16HBE cells or H1975 cells transduced with PCHD; ##*p* < 0.01 and ###*p* < 0.001 versus H1975 cells transduced with sh‐NC. Cell experiment was repeated three times.

MELK can curb the ubiquitination of EZH2 by phosphorylation, thus promoting the stability of EZH2 protein.^16^ GEPIA data analysis manifested that EZH2 was upregulated in LUAD tissues (Figure 3A).

Then, the effect of MELK on EZH2 expression in LUAD cells was explored. Immunoblotting and RT‐qPCR exhibited that compared with 16HBE cells, EZH2 expression was prominently enhanced (Figure 3B). In addition, overexpression of MELK elevated the EZH2 protein level, while downregulation of MELK reduced the EZH2 protein level in (Figure 3D), but had little effect on the mRNA level of EZH2 (Figure 3C). In summary, MELK might manipulate EZH2 expression at the protein level in LUAD cells.

Co‐IP experiment displayed that MELK interacted with EZH2 (Figure 3E). Further, CHX tracing experiment showed that overexpression of MELK augmented the stability of EZH2 and lowered its degradation rate (Figure 3F). Silencing of MELK enhanced the stability of EZH2 and accelerated its degradation rate (Figure 3G). Meanwhile, the addition of proteasome inhibitor MG132 reduced the ubiquitination of EZH2 protein after silencing MELK (Figure 3H). Together, MELK could interact with EZH2, which inhibited the ubiquitination of EZH2 in LUAD cells.

### 3.4 EZH2 promotes H3K27me3 modification of LATS2 promoter

**FIGURE 4:**
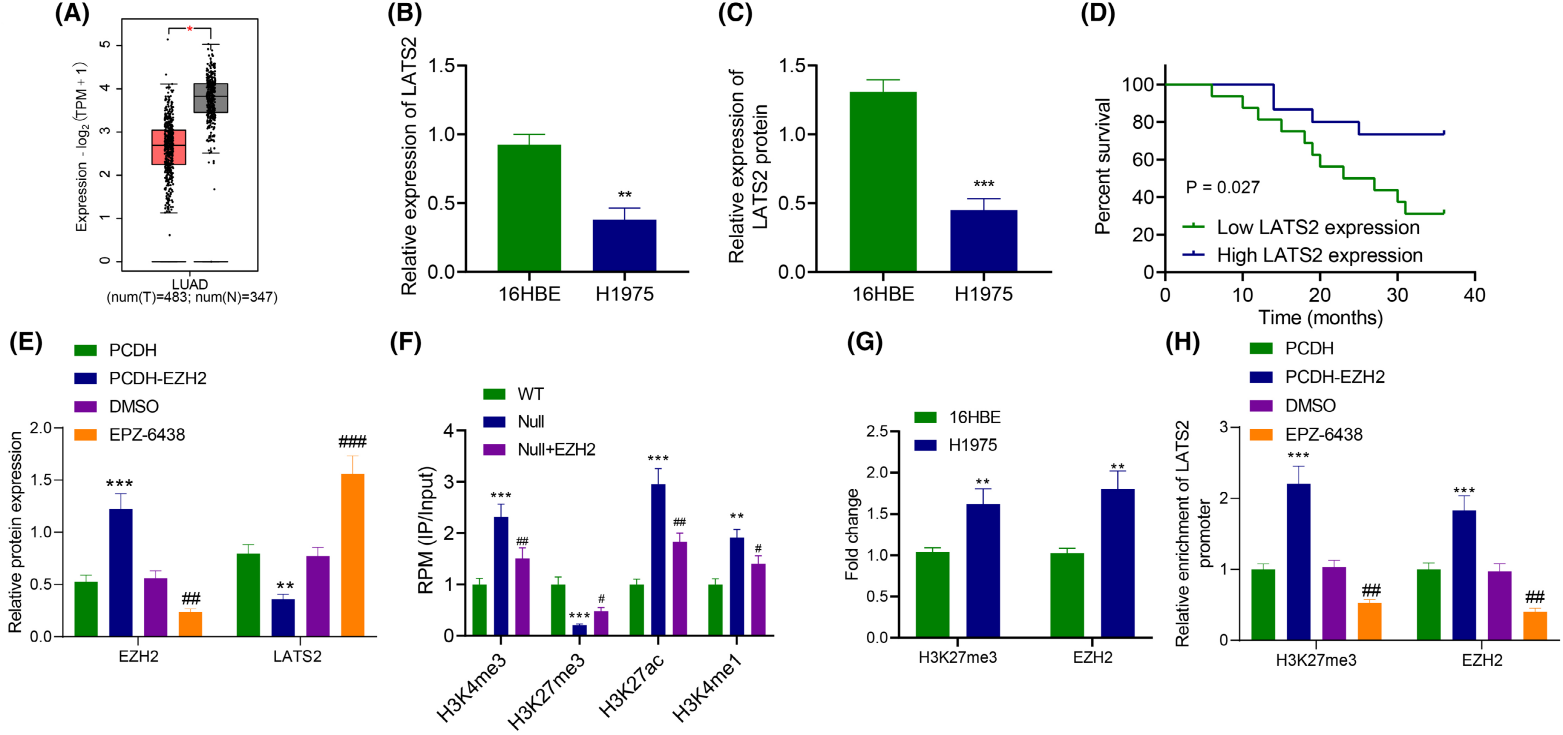
EZH2 regulates H3K27me3 modification in LATS2 promoter to reduce LATS2 expression in LUAD cells. (A)LATS2 mRNA level in GEPIA database. Red indicates tumour tissue and grey indicates normal tissues (*p < 0.05 vs. normal tissues). (B) mRNA level of LATS2 in 16HBE and H1975 cells detected by RT‐qPCR. (C) Protein level of LATS2 in 16HBE and H1975 cells measured by western blot analysis. (D) The correlation between LATS2 expression and OS of LUAD patients analysed by Kaplan–Meier method. (E) Protein levels of EZH2 and LATS2 in H1975 cells after overexpression or inhibition of EZH2 detected by western blot analysis. (F) Expression of H3K27me3, H3K27ac, H3K4me1 and H3K4me3 in LATS2 promoter region after EZH2 silencing in ChIP‐seq GSE76626 of human embryonic stem cells. (G) The enrichment of H3K27me3 and EZH2 in the promoter region of LATS2 in 16HBE and H1975 cells detected by ChIP assay. H, The enrichment of H3K27me3 and EZH2 in LATS2 promoter region of H1975 cells after overexpression or inhibition of EZH2 measured by ChIP assay. **p < 0.01 and ***p < 0.001 versus 16HBE cells or H1975 cells transduced with PCHD; #*p* < 0.05, ##*p* < 0.01 and ###*p* < 0.001 versus H1975 cells treated with DMSO. The cell experiment was repeated three times.

It has been documented that EZH2 is enriched in the promoter region of LATS2 and regulates its expression via H3K27me3.^12^ Differential analysis of GEPIA data exhibited that LATS2 expression was decreased in LUAD (Figure 4A).

Immunoblotting and RT‐qPCR depicted that compared with 16HBE cells, LATS2 expression was diminished (Figure 4B,C). As reflected by Kaplan–Meier, the OS of patients with low LATS2 expression was prominently lower than those of patients with high LATS2 expression (Figure 4D).

EZH2 was overexpressed to verify whether EZH2 was enriched in the promoter region of LATS2 and affected its expression through H3K27me3. Based on immunoblotting results, overexpression of EZH2 reduced LATS2 expression, while it was also prominently elevated following treatment with EZH inhibitor EPZ‐6438 (Figure 4E). Then, ChIP‐seq microarray data GSE76626 from the GEO database was selected to analyse the expression of H3K27me3, H3K27ac, H3K4me1 and H3K4me3 in the LATS2 promoter region after EZH2 silencing. The results documented that after silencing EZH2, H3K27me3 level was decreased in the LATS2 promoter region, but H3K27ac, H3K4me1 and H3K4me3 expression exhibited no noticeable difference (Figure 4F).

ChIP found that EZH2 or H3K27me3 antibody enrichment in the LATS2 promoter increased when compared with HEB cells (Figure 4G). In addition, the enrichment of EZH2 and H3K27me3 in the promoter region of LATS2 elevated after overexpression of EZH2, while opposing findings were detected after treatment with EPZ‐6438 (Figure 4H). In conclusion, EZH2 inhibited LATS2 expression by mediating H3K27me3.

### 3.5 MELK induces malignant properties of LUAD cells via the EZH2/LATS2 axis

**FIGURE 5:**
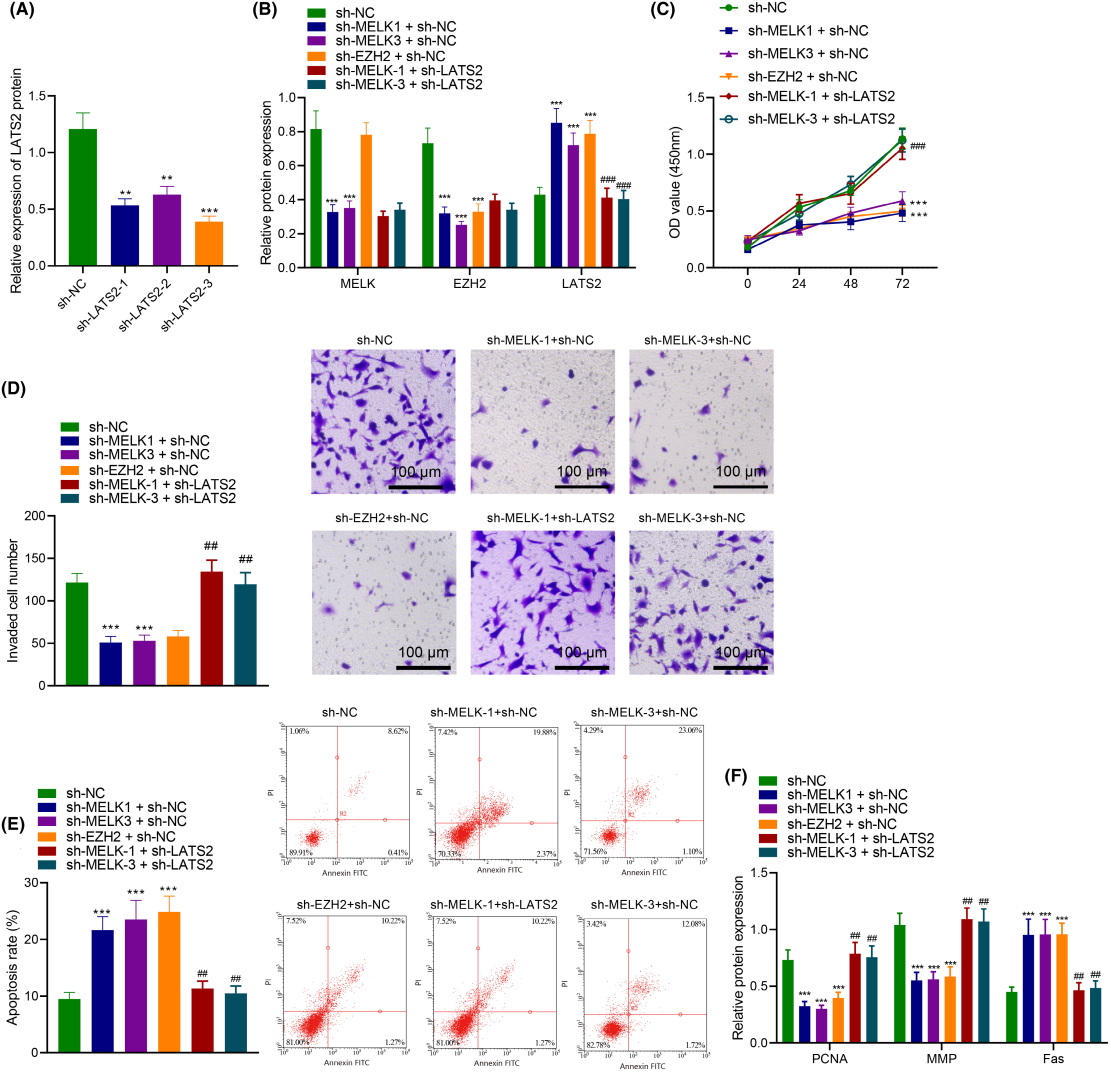
MELK knockdown depresses LUAD cell proliferation and invasion but accelerates apoptosis via the EZH2/LATS2 axis. (A) The expression of LATS2 in H1975 cells transduced with sh‐LATS2‐1, sh‐LATS2‐2 or sh‐LATS2‐3 measured by western blot analysis. H1975 cells were transduced with sh‐NC, sh‐MELK + sh‐NC, sh‐EZH2 + sh‐NC or sh‐MELK + sh‐LATS2. (B) MELK, EZH2 and LATS2 protein levels in H1975 cells measured by western blot analysis. (C) Viability of H1975 cells detected by CCK‐8 assay. (D) Invasion of H1975 cells detected by Transwell assay. (E) Apoptosis of H1975 cells detected by flow cytometry. (F) Expression of proliferation, invasion and apoptosis markers (PCDA, MMP and Fas) in H1975 cells measured by western blot analysis. **p < 0.01 and ***p < 0.001 versus H1975 cells transduced with sh‐NC; ##*p* < 0.01 and ###*p* < 0.001 versus H1975 cells transduced with sh‐MELK + sh‐NC. The cell experiment was repeated three times.

According to the above conclusions, we proposed a hypothesis that MELK mediates the H3K27me3 modification of LATS2 by affecting the expression of EZH2, which affects the proliferation, invasion and apoptosis of LUAD cells. To verify this hypothesis, we constructed LATS2 interference plasmids and transfected them into H1975 and PC‐9 cells. Western blot analysis showed that the expression of LATS2 was significantly decreased in sh‐LATS2‐1, sh‐LATS2‐2 and sh‐LATS2‐3 groups compared to the sh‐NC group, with sh‐LATS2‐3 having the lowest expression level. Therefore, sh‐LATS2‐3 was used for subsequent experiments and named sh‐LAST2 (Figure 5A).

Next, we transfected different plasmids (sh‐NC, sh‐MELK‐1 + sh‐NC, sh‐MELK‐3 + sh‐NC, sh‐EZH2 + sh‐NC and sh‐MELK+sh‐LATS2) into H1975 and PC‐9 cells. The western blot results showed that compared to the sh‐NC group, the MELK and EZH2 expression levels decreased, and the LATS2 expression level increased in the sh‐MELK‐1 + sh‐NC and sh‐MELK‐3 + sh‐NC groups. In the sh‐EZH2 + sh‐NC group, the MELK expression level did not change, the EZH2 expression level decreased and the LATS2 expression level increased. Compared with the sh‐MELK‐1 + sh‐NC group, the expression levels of MELK and EZH2 did not change in the sh‐MELK‐1 + sh‐LATS2 group, but the LATS2 expression level significantly decreased. Compared with the sh‐MELK‐3 + sh‐NC group, the expression levels of MELK and EZH2 did not change in the sh‐MELK‐3 + sh‐LATS2 group, but the LATS2 expression level significantly decreased (Figure 5B).

Furthermore, we found, through CCK8, Transwell and flow cytometry assays, that compared with the sh‐NC group, the proliferation and invasion abilities significantly decreased, and the apoptosis rate was significantly increased in the sh‐MELK‐1 + sh‐NC, sh‐MELK‐3 + sh‐NC and sh‐EZH2 + sh‐NC groups. Compared with the sh‐MELK‐1 + sh‐NC group, the proliferation and invasion abilities significantly increased, and the apoptosis rate significantly decreased in the sh‐MELK‐1 + sh‐LATS2 group. Compared with the sh‐MELK‐3 + sh‐NC group, the proliferation and invasion abilities significantly increased, and the apoptosis rate significantly decreased in the sh‐MELK‐3 + sh‐LATS2 group (Figure 5C–E). In addition, we also found that compared with the sh‐NC group, the PCNA and MMP levels significantly decreased, and Fas levels were significantly increased in the sh‐MELK‐1 + sh‐NC, sh‐MELK‐3 + sh‐NC and sh‐EZH2 + sh‐NC groups. Compared with the sh‐MELK‐1 + sh‐NC group, the PCNA and MMP levels significantly increased, and Fas levels significantly decreased in the sh‐MELK‐1 + sh‐LATS2 group. Compared with the sh‐MELK‐3 + sh‐NC group, the PCNA and MMP levels significantly increased, and Fas levels significantly decreased in the sh‐MELK‐3 + sh‐LATS2 group (Figure 5F).

These results suggest that MELK inhibits the expression of LATS2 through EZH2, thereby promoting the proliferation and invasion of LUAD cells and inhibiting their apoptosis.

### 3.6 MELK orchestrates EZH2/LATS2 axis to promote tumour formation of LUAD cells in vivo

**FIGURE 6:**
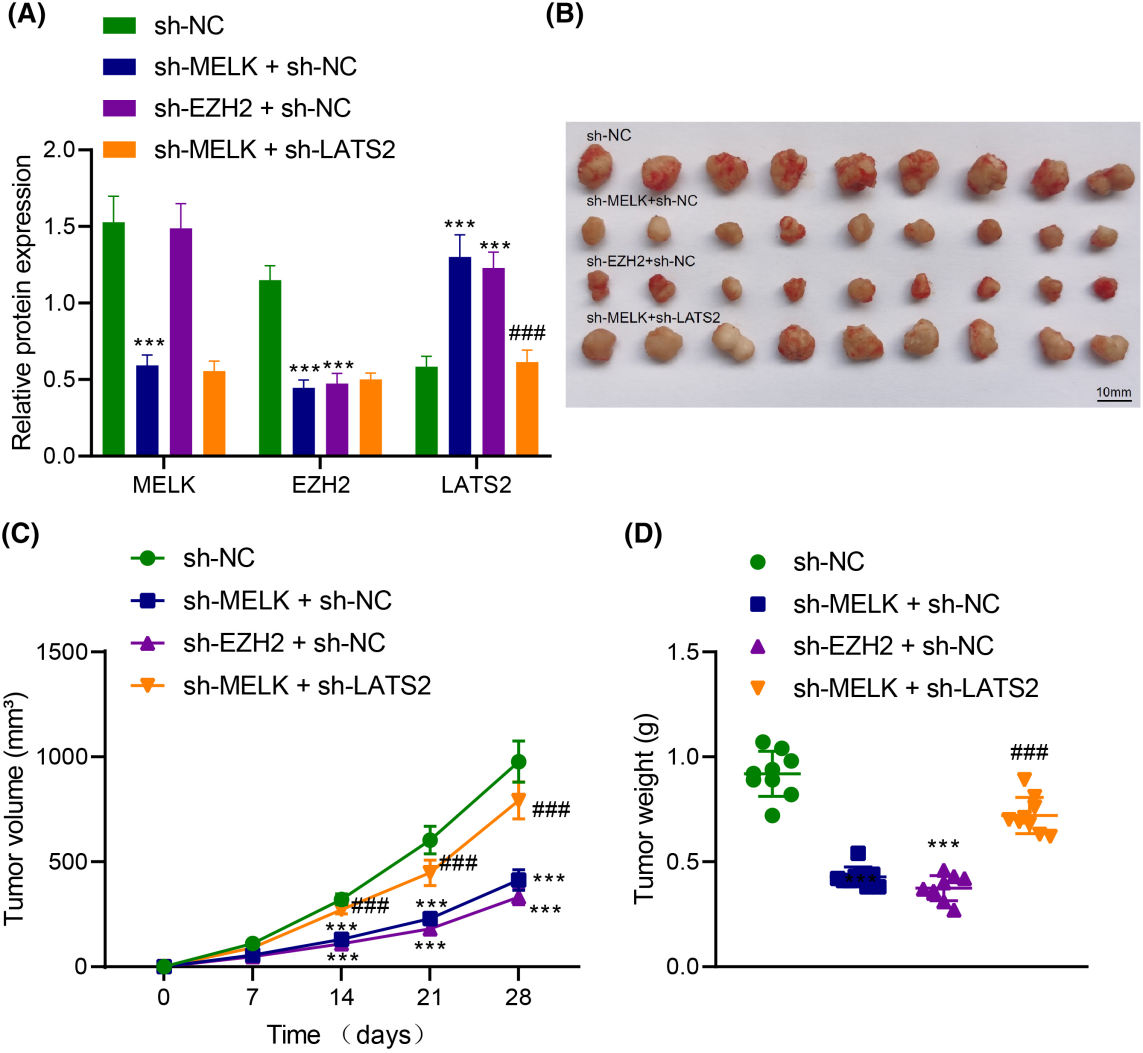
MELK accelerates the tumorigenicity of LUAD cells in nude mice via the EZH2/LATS2 axis. Nude mice were injected with H1975 cells transduced with sh‐NC, sh‐MELK + sh‐NC, sh‐EZH2 + sh‐NC or sh‐MELK + sh‐LATS2 (*N* = 9). (A) The expression of MELK, EZH2 and LATS2 in tumour tissues of nude mice evaluated by western blot analysis. (B) Representative images of tumours of nude mice. (C) Tumour volume in nude mice. (D) Tumour weight in nude mice. ***p < 0.001 versus nude mice injected with H1975 cells transduced with sh‐NC; ###*p* < 0.001 versus nude mice injected with H1975 cells transduced with sh‐MELK + sh‐NC.

To investigate the effect of MELK regulating the EZH2/LATS2 axis on the tumorigenicity of LUAD cells in vivo, nude mice were injected with H1975 cells with different treatments. According to immunoblotting, lowered MELK and EZH2 expression and elevated LATS2 expression were observed in tumour tissues of nude mice injected with H1975 cells transduced with sh‐MELK. MELK expression remained unchanged, EZH2 expression decreased and LATS2 expression was augmented in tumour tissues of nude mice injected with H1975 cells transduced with sh‐EZH2. Both depletion of MELK and EZH2 exerted no effects on MELK and EZH2 expression but reduced LATS2 expression in tumour tissues of nude mice (Figure 6A). In addition, MELK knockdown or downregulated EZH2 reduced the weight and volume of subcutaneous tumours of nude mice. Both downregulation of MELK and EZH2 contributed to enhancing the weight and volume of subcutaneous tumours in nude mice (Figure 6B–D).

## 4 DISCUSSION

**FIGURE 7:**
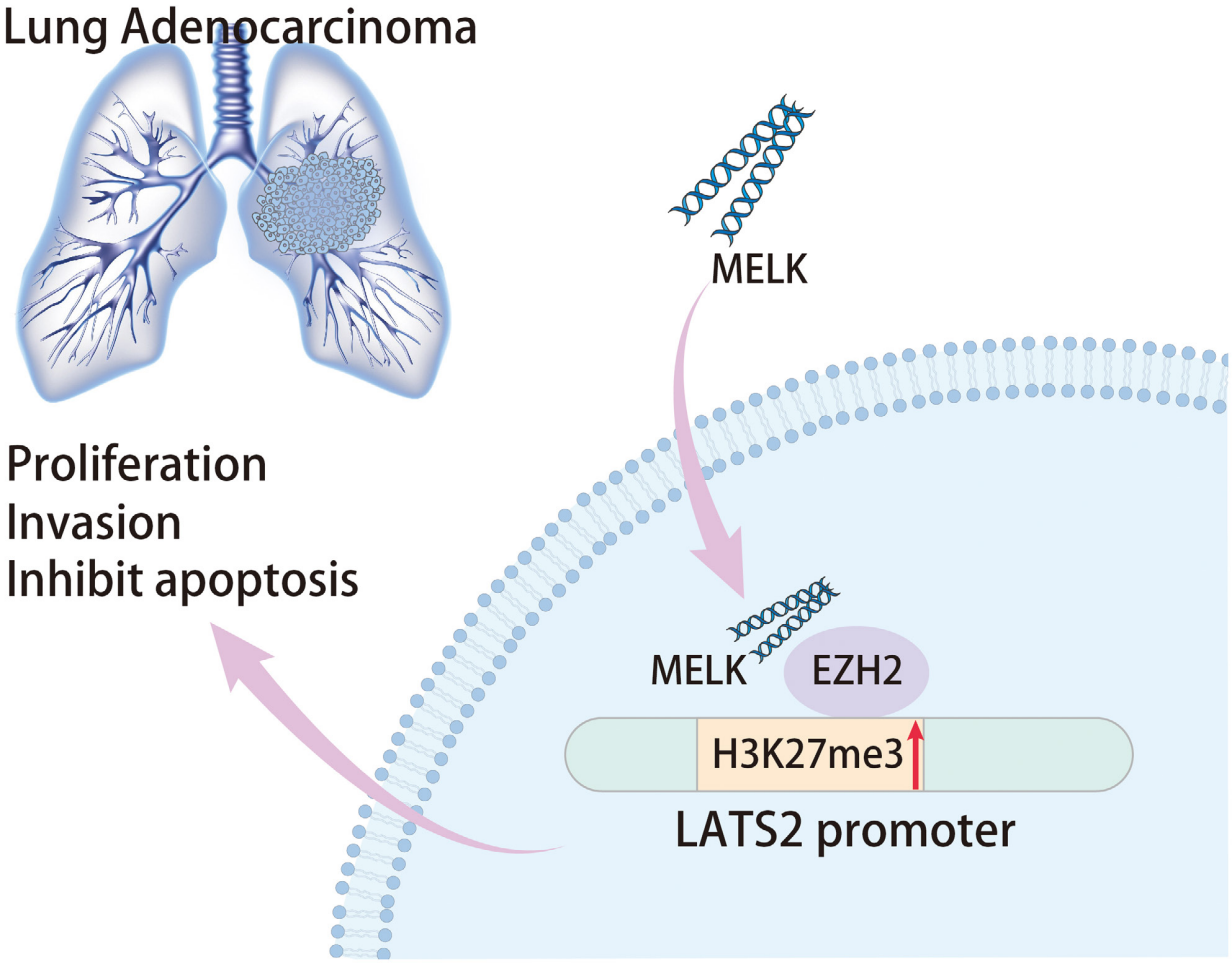
Mechanism of MELK regulating H3K27me3 modification of LATS2 through EZH2 in the occurrence and development of LUAD. MELK might cause EZH2 upregulation by inducing its ubiquitination and degradation, leading to the increase of H3K27me3 modification in the LATS2 gene promoter, thus facilitating the tumorigenicity of LUAD.

LUAD remains a major cause of mortality related to cancer around the world.^17^ Besides, the existing literature has reported that MELK is implicated in numerous human cancers, including LUAD.^18^ However, the mechanism of MELK in LUAD remains to be further ascertained. In this context, this research was designed to elucidate the mechanism of MELK behind LUAD. The findings of our study indicated that MELK might cause EZH2 upregulation by inducing its ubiquitination and degradation, leading to the increase of H3K27me34 modification in the LATS2 gene promoter, thus facilitating tumorigenicity of LUAD (Figure 7).

In our investigation, MELK was highly expressed in LUAD tissues and cells, and MELK silencing resulted in the inhibition of the proliferation and invasion and facilitation of apoptosis of LUAD cells with reduced expression of PCNA and MMP and elevated Fas expression. PCNA, MMP and Fas are indicators of cell proliferation, invasion and apoptosis, respectively, which are expressed in LUAD tissues.^19^, ^20^, ^21^ Consistently, Xie et al. have observed that MELK is upregulated in LUAD, associated with a poor prognosis of LUAD patients.^22^ Another work has also illustrated that MELK knockdown suppresses migration and invasion of LUAD cells, indicating a potential therapeutic target for LUAD.^18^ In addition, downregulation of MELK suppresses proliferation and survival of small cell lung cancer (SCLC) cells in vitro and tumour growth in vivo.^23^ These findings support that MELK acted as an oncogene in LUAD.

It was previously displayed that MELK upregulation augmented the stability of EZH2 through inhibition of EZH2 ubiquitination in extranodal natural killer/T‐cell lymphoma cells. Suppression of MELK via both chemical and genetic methods resulted in ubiquitination and destabilization of EZH2 protein.^16^ Similarly, our finding also elaborated that MELK promoted EZH2 ubiquitination and degradation to stabilize EZH2 protein in LUAD cells. Interestingly, MELK mediates EZH2 phosphorylation to promote the progression of human cancers, such as glioma.^24^ Another crucial result in this study uncovered that EZH2 ectopic expression enhanced LUAD cell proliferative, invasive and migrating potentials but restrained their apoptosis. The tumour‐promoting properties of EZH2 have been demonstrated in various cancers, like ovarian, prostate and breast cancers.^25^, ^26^, ^27^ Furthermore, a prior study has discovered that EZH2 expression is increased in LUAD, which is related to poor prognosis in LUAD patients,^28^ which is consistent with our finding. Besides, our findings also agree with a previous report that reduced EZH2 expression contributes to the decline in the growth and metastasis of LUAD cells.^13^ Hence, MELK silencing downregulated EZH2 to inhibit LUAD cell proliferation and invasion and induce apoptosis, thereby suppressing tumour growth.

As widely recognized, EZH2 is the enzymatic catalytic subunit of PRC2 capable of modulating gene expression via H3K27me3.^29^ For example, the research conducted by Tang et al. found that EZH2‐mediated H3K27me3 in the enhancer regions inhibited CCL2 expression, thereby inducing tumour development in SCLC.^30^ Intriguingly, our obtained data deciphered that silencing EZH2 curtailed H3K27me3 modification on the LATS2 gene promoter to elevate LATS2 expression in LUAD cells. Similarly, EZH2 can be enriched in the LATS2 promoter region to affect its expression through H3K27me3 in fulminant hepatic failure.^12^ In this study, LATS2 is downregulated in LUAD tissues and cells, and depleted LATS2 facilitated cell invasion, migration and proliferation but restrained apoptosis in LUAD. It has been documented that LATS2 is a widely recognized anti‐oncogene in numerous cancers by regulating tumour cell proliferation and apoptosis.^31^, ^32^, ^33^ Corroborating findings are reported in the existing research that LATS2 is poorly expressed in NSCLC, which is related to the poor prognosis of patients with NSCLC.^34^ Notably, a recent study has also revealed that overexpression of LATS2 can inhibit malignant features of lung cancer cells, suggesting that LATS2 acts as a tumour suppressor in lung cancer.^35^ The suppressive effects of LATS2 on LUAD have also been reported.^14^ Therefore, the downregulation of MELK may restrain LUAD by suppressing EZH2 and upregulating LATS2.

Conclusively, our data provided a novel insight into the oncogenic mechanism of MELK in LUAD by facilitating cell malignant properties, which was achieved by the upregulation of EZH2 and the downregulation of LATS2. The discovery of MELK as a mediator that orchestrates the EZH2‐LATS2 axis in LUAD offers new insight into LUAD treatment.

## AUTHOR CONTRIBUTIONS

**Hui Yu:** Formal analysis (equal); resources (equal); writing – original draft (equal); writing – review and editing (equal). **Xianrong Xu:** Writing – original draft (equal); writing – review and editing (equal). **Lirong Zhu:** Writing – original draft (equal); writing – review and editing (equal). **Shengjie Chen:** Data curation (equal); writing – review and editing (equal). **Jincheng He:** Data curation (equal); writing – review and editing (equal).

## FUNDING INFORMATION

Not applicable.

## CONFLICT OF INTEREST STATEMENT

The authors declare that they have no competing interests.

## CONSENT FOR PUBLICATION

Not applicable.

## Supporting information


Figure S1

Table S1

Table S2

## Data Availability

The original contributions presented in this study are included in the article/supplementary materials; further inquiries can be directed to the corresponding author.
