# Supplementary figures and images for: MELK aggravates lung adenocarcinoma by regulating EZH2 ubiquitination and H3K27me3 histone methylation of LATS2
Source: J Cell Mol Med. 2024 Apr 23;28(8):e18216. doi: 10.1111/jcmm.18216 (PMC11037405; doi:10.1111/jcmm.18216)

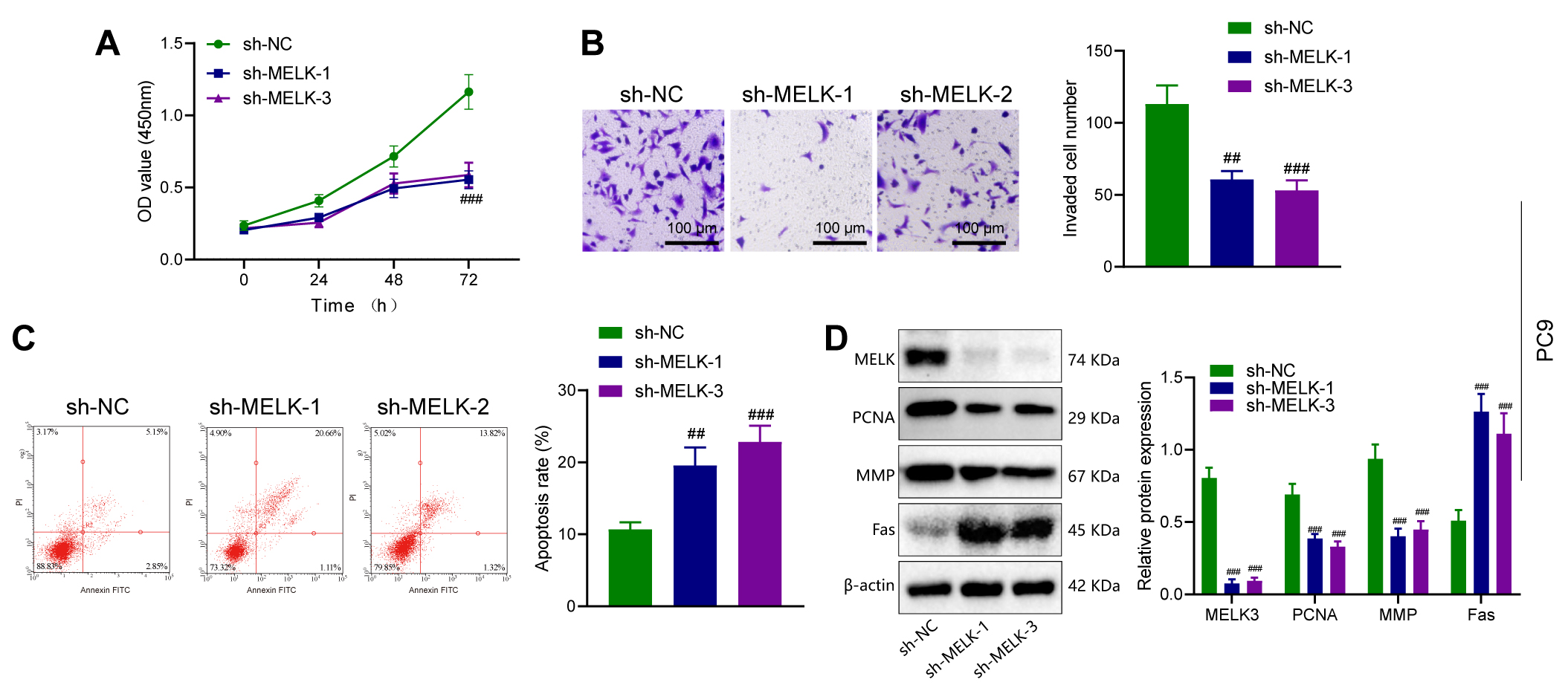

Supplement: Supplementary file 1 — Figure S1 [file JCMM-28-e18216-s003.jpg]
