# Supplementary material for: MELK aggravates lung adenocarcinoma by regulating EZH2 ubiquitination and H3K27me3 histone methylation of LATS2
Source: J Cell Mol Med. 2024 Apr 23;28(8):e18216. doi: 10.1111/jcmm.18216 (PMC11037405; doi:10.1111/jcmm.18216)
Supplement: Supplementary file 2 — Table S1 [file JCMM-28-e18216-s002.docx]

**Supplementary Table 1** The silencing plasmids information

| Group | Sequence |
| --- | --- |
| sh-NC | 5'-TTCTCCGAACGTGTCACGTTT-3' |
| sh-MELK-1 | 5'-GCTTTGCAAAGGTCAAACTTG-3' |
| sh-MELK-2 | 5'-GCAAAGGTCAAACTTGCCTGC-3' |
| sh-MELK-3' | 5'-GGTCAAACTTGCCTGCCATAT-3' |
| sh-EZH2-1 | 5'-GGATGGTACTTTCATTGAAGA-3' |
| sh-EZH2-2 | 5'-GGTGAATGCCCTTGGTCAATA-3' |
| sh-EZH2-3 | 5'-GCAGCTTTCTGTTCAACTTGA-3' |
| sh-LATS2-1 | 5'-GCCACGACTTATTCTGGAAAT-3' |
| sh-LATS2-2 | 5'-GGACCAAACAGTGACACTTCC-3' |
| sh-LATS2-3 | 5'-GCAGATTGTGCGGGTCATTAA-3' |
