# Supplementary material for: MELK aggravates lung adenocarcinoma by regulating EZH2 ubiquitination and H3K27me3 histone methylation of LATS2
Source: J Cell Mol Med. 2024 Apr 23;28(8):e18216. doi: 10.1111/jcmm.18216 (PMC11037405; doi:10.1111/jcmm.18216)
Supplement: Supplementary file 3 — Table S2 [file JCMM-28-e18216-s001.docx]

**Supplementary Table 2** The primers for RT-qPCR

| Gene | Sequences |
| --- | --- |
| MELK | Forward: 5'-ACTGCCCTGGAGGAGAGCTG-3' |
|  | Reverse: 5'-AGCCCTGGCTGTGCACATAA-3' |
| EZH2 | Forward: 5'-ACGGCTTCCCAATAACAGTA-3' |
|  | Reverse: 5'-TGTTTGACACCGAGAATTTGC-3' |
| LATS2 | Forward: 5'-AGATTTCGGCCTCTGCACTG-3' |
|  | Reverse: 5'-TAGGGTCTTCAGCCTGTCCC-3' |
| β-actin | Forward: 5'-CACTGTGCCCATCTACGAGG-3' |
|  | Reverse: 5'-TAATGTCACGCACGATTTCC-3' |
